# Evolutionary Adaptation of the Amino Acid and Codon Usage of the Mosquito Sodium Channel following Insecticide Selection in the Field Mosquitoes

**DOI:** 10.1371/journal.pone.0047609

**Published:** 2012-10-17

**Authors:** Qiang Xu, Lee Zhang, Ting Li, Lan Zhang, Lin He, Ke Dong, Nannan Liu

**Affiliations:** 1 Department of Entomology and Plant Pathology, Auburn University, Auburn, Alabama, United States of America; 2 Genomics Laboratory, Auburn University, Auburn, Alabama, United States of America; 3 Department of Entomology, Michigan State University, East Lansing, Michigan, United States of America; National Institute of Allergy and Infectious Diseases, United States of America

## Abstract

Target site insensitivity resulting from point mutations within the voltage-gated sodium channel of the insect nervous system is known to be of primary importance in the development of resistance to pyrethroid insecticides. This study shifts current research paradigms by conducting, for the first time, a global analysis of all the naturally occurring mutations, both nonsynonymous and synonymous mutations, as well as mutation combinations in the entire mosquito sodium channel of *Culex quinquefasciatus* and analyzing their evolutionary and heritable feature and roles in insecticide resistance. Through a systematic analysis of comparing nucleotide polymorphisms in the entire sodium channel cDNAs of individuals between susceptible and resistant mosquito strains, between field parental mosquitoes and their permethrin selected offspring, and among different mosquito groups categorized by their levels of tolerance to specific permethrin concentrations within and among the mosquito strains of the field parental strains and their permethrin selected offspring, 3 nonsynonymous (A^109^S, L^982^F, and W^1573^R) and 6 synonymous (L^852^, G^891^, A^1241^, D^1245^, P^1249^, and G^1733^) mutations were identified. The co-existence of all 9 mutations, both nonsynonymous and synonymous, and their homozygousity were found to be important factors for high levels of resistance. Our study, for the first time, provide a strong case demonstrating the co-existence of both nonsynonymous and synonymous mutations in the sodium channel of resistant mosquitoes in response to insecticide resistance and the inheritance of these mutations in the offspring of field mosquito strains following insecticide selection.

## Introduction

Vector control of mosquitoes is an important part of the current global strategy to control mosquito-associated diseases. Insecticides are the most important component in the vector-control effort, of which pyrethroids are currently the most widely used for indoor spraying of mosquitoes worldwide. However, the widespread growth of resistance to insecticides in mosquitoes, especially to pyrethroids, is rapidly becoming a global problem, resulting in the rise of mosquito-borne diseases [1]. The voltage- gated sodium channel is the primary target of both pyrethroids and DDT. Modifications in the sodium channel structure (specifically, point mutations resulting from single nucleotide polymorphisms [SNPs]), lead to insensitivity of insect sodium channels to pyrethroids and DDT and result in the development of insecticide resistance, known as knockdown resistance (kdr) [2]–[5].

Over the past decade, studies have provided evidence for the involvement of point mutations (*kdr* mutations) in voltage-gated sodium channels in kdr-like resistance of many insect species [3]–[8]. Among these *kdr* mutations, substitution of leucine to phenylalanine [L to F], histidine [L to H], or serine [L to S] in the 6^th^ segment of domain II (IIS6) has been clearly associated with resistance to pyrethroids and DDT in many insect species, including mosquitoes [9]–[12] while other *kdr* mutations appeared to be unique to specific species [3]–[5]. Systematic *in vitro* site-directed mutagenesis in insect sodium channel genes has revealed multiple regions of sodium channels that contribute to the binding and action of pyrethroids [13], [14], suggesting that the interaction of multiple mutations may play a role in the response of an insect sodium channel to insecticides. However, to date, research on insecticide resistance and insect sodium channels has focused primarily on nonsynonymous mutations in the sodium channel and little work has been done on the potential contribution of synonymous mutations to insecticide resistance in insects.

The research reported here shifts current research paradigms by conducting, for the first time, a global analysis of all the naturally occurring mutations, both nonsynonymous and synonymous mutations, as well as mutation combinations in the entire mosquito sodium channel of the field parental strain and its permethrin selected offspring of *Culex quinquefasciatus*; characterizing the co-occurrence of both nonsynonymous and synonymous mutations in insecticide-resistant mosquitoes and their inheritance flowing the insecticide selection; and determining the specific threshold of insecticide concentrations at which particular mutations or mutation combinations occur in a mosquito strain or group. One of the principal questions addressed by this study was the association of synonymous mutations with insecticide resistance in mosquitoes and their possible role in resistance.

## Materials and Methods

### Mosquito Strains of *Cx. quinquefasciatus*

Mosquito strains of S-Lab, S-Lab^G5^, HAmCq^G0^ and HAmCq^G8^ were used in the study. The S-Lab^G5^ was the S-Lab strain that had been selected with permethrin for 5 generations. The concentration used in all 5 generation selections was 0.005–0.006 ppm with 30 to 35% percent survivors each generation and the level of tolerance to permethrin in each permethrin selected generation did not change, i.e., the S-Lab^G1^ to S-Lab^G5^ had the similar levels of susceptibility to permethrin as that in S-Lab (Table S1). Thus, S-Lab^G5^ was used as a reference susceptible strain in the study. HAmCq^G0^ was the field parental resistant strain with more than 70 egg rafts collected from one location of Huntsville, Alabama, USA in 2002 [15]–[17]. HAmCq^G0^ was established in laboratory without further exposure to insecticides. The resistance level to permethrin in HAmCq^G0^ was 10-fold compared with S-Lab [18]. The HAmCq^G8^ strain was the 8^th^ generation of permethrin-selected HAmCq^G0^ offspring and had a 2,700-fold level of resistance [18]. HAmCq^G8^ was maintained in the laboratory under biannual selection with permethrin. The resistance levels on these mosquito strains were re-measured every three months.

### Amplification of the Full Length of Sodium Channel cDNAs in *Cx. quinquefasciatus*

First strand cDNA was synthesized with SuperScript II reverse transcriptase (Invitrogen) and an antisense 5¢-anchored oligo(dT) primer (Table S2) using the mRNAs as templates. The partial cDNA fragments of the mosquito sodium channel gene were amplified by polymerase chain reaction (PCR). The PCR solution with cDNA template and a primer pair, PG KDR S4 and KDR AS02 (Table S2, 19) were heated to 94°C for 2 min, followed by 40 cycles of PCR reaction (94°C for 45 s, 60°C for 45 s and 72°C for 3 min) and a final extension of 72°C for 10 min.

RACE was then carried out using the Marathon^Tm^ cDNA Amplification Kit (Clontech) [19]. The first strand cDNAs were synthesized with AMV reverse transcriptase using *Culex* mosquito mRNAs. The double strand cDNA was subsequently synthesized and adaptors were ligated to both ends of each double strand cDNA using T4 DNA ligase as described by the manufacturer (Clontech). The 5' and/or 3' ends of the sodium channel cDNA fragments were amplified by PCR using adapter primer AP1 and gene specific primers, KDR AS 34 and KDR S03, (Table S2) generated based on the 5' and/or 3' end sequences of the partial sodium channel cDNA fragments, respectively. The PCR reaction was heated to 95°C for 5 min followed by 35 cycles of 94°C for 1 min, 58°C for 1 min, and 68°C for 4 min with a final extension step at 72°C for 10 min.

The full length of the *Cx. quinquefasciatus* sodium channel cDNA was subsequently isolated for each of mosquito strains by RT-PCR using the Expand Long Range, dNTPack kit (Roche) with a specific primer pair, KDR S16/KDR AS09 (Table S2), synthesized based on the respective 5' and 3' end sequences of the putative sodium channel genes. The PCR reaction was conducted following a PCR cycle of 92°C for 2 min, 10 cycles of 92°C for 10 s, 55°C for 15 s, and 68°C for 6 min, and 35 cycles of 92°C for 10 s, 55°C for 15 s, and 68°C for 6 min and 20 s, with a final extension of 68°C for 10 min. All PCR products were cloned into PCR™ 2.1 Original TA cloning vector (Invitrogen) and sequenced. Cloning and sequence analyses of sodium channel cDNA fragments were repeated at least three times for each mosquito strain with different preparations of mRNAs. Three TA clones from each replication were sequenced, hence a total of 9 complete sodium channel cDNA sequences were analyzed for each mosquito strain.

### Permethrin Treatment

Preliminary concentration ranges for larvae were performed, generating corresponding concentration ranges of LC_10_, LC_50_, and LC_90_ for each mosquito strain (Table 1), which were used to treat each of HAmCq^G0^and HAmCq^G8^, generating 8 larval groups with different levels of resistance to permethrin insecticide. About1500 4^th^ instar larvae of each *Culex* strain were treated with permethrin at their respective LC_10_ concentrations. Eight hours after this treatment, the dead mosquitoes were collected as group 1 of each mosquito strain (i.e., HAmCq^G0^-<LC_10_ or HAmCq^G8^-<LC_10_). The surviving mosquitoes were then exposed to permethrin LC_50_ concentrations. Eight hours after this treatment, the dead mosquitoes were collected as group 2 of each mosquito strain (HAmCq^G0^-LC_10–50_, or HAmCq^G8^-LC_10–50_). The surviving mosquitoes from the permethrin LC_50_ concentration treatment were then exposed to permethrin LC_90_ concentrations. Eight hours after treatment, the dead and surviving mosquitoes were separately collected as group 3 (HAmCq^G0^-LC_50–90_ or HAmCq^G8^-LC_50–90_) and group 4 (HAmCq^G0^->LC_90_ or HAmCq^G8^->LC_90_), respectively. Each treatment was repeated 2 times. In this study, the critical criterion was that only individuals that had all 9 mutations tested could be utilized for the data analyses. Data from a total of 40 individual mosquitoes that met this criterion in each of the 16 groups were collected and analyzed.

**Table 1:** Permethrin treatment of *Culex* mosquitoes and mosquito groups generated after treatment.

	Permethrin Treatments*									
	LC_10_ Treatment			LC_50_ Treatment			LC_90_ Treatment			
Strain	n‡	†LC_10_	1^st^ Group	n§	†LC_50_	2^nd^ Groups	n¶	†LC_90_	3^rd^ Groups	4^th^ Groups
		PPM	(collect dead mosquitoes)		PPM	(collect dead mosquitoes)		PPM	(collect dead mosquitoes)	(collect alive mosquitoes)
HAmCq^G0^	∼1500	0.005	HAmCq^G0^-<LC_10_	∼1300	0.05	HAmCq^G0^-LC_10–50_	∼700	0.2	HAmCq^G0^-LC_50–90_	HAmCq^G0^->LC_90_
HAmCq^G8^	∼1500	2	HAmCq^G8^-<LC_10_	∼1300	30	HAmCq^G8^-LC_10–50_	∼700	60	HAmCq^G8^-LC_50–90_	HAmCq^G8^->LC_90_

* Each treatment was repeated 3 times.

‡ The total number of early 4^th^ instar larvae used at the beginning of the permethrin treatment with LC_10_ for each replication.

† The concentrations of permethrin to these mosquitoes have been identified previously [10], [18].

§ The mosquitoes surviving from permethrin treatment with LC_10_ 10 h after treatment.

¶ The mosquitoes surviving from the permethrin treatment with LC_50_ 10 h after treatment.

### Single Nucleotide Polymorphism (SNP) Determination of the Mutations

SNP determination was conducted on both adults of each of mosquito strains and 4^th^ instar larvae of each of permethrin treated groups using an ABI Prism SNaPshot Multiplex Kit and data were analyzed on the ABI Prism® 3100 Genetic Analyzer using Genemapper software according to the manufacture’s instructions (A&B Applied Biosystems). Three replications were performed for each of adult experiments and a total of 60 individual mosquitoes were used for each strain with 20 (10 males and 10 females) for each replication. Whereas, two replications were performed for each 4^th^ instar larvae experiment and a total of 40 individual 4^th^ instar larvae were used for each of permethrin treated groups with 20 for each replication. The first strand cDNAs were synthesized from each individual mosquito using the oligo(dT) primer as described above. Three PCR primer pairs, KDR S16/KDR AS34, PG_KDR S4/KDR AS02, and KDR S03/KDR AS09 (Table S2) were designed according to the specific sequences of full length *Culex* sodium channel cDNAs (Fig. S1) to amplify three sodium channel cDNA fragments from each of the individual mosquitoes on which the polymorphisms reside. Each PCR reaction with the cDNA template and a primer pair was heated to 94°C for 2 min, followed by 40 cycles of PCR reaction (94°C for 45 s, 60°C for 45 s and 72°C for 3 min) and a final extension of 72°C for 10 min. PCR products were used as the templates for the SNP determination. Each PCR reaction was performed 3 times on the cDNA of each of a total of 40 individuals of 4^th^ instar larvae from each of the mosquito groups or 60 individuals of 3 day old adults from each mosquito strain. The PCR products served as the replication for the SNP determination of each polymorphism. Three replications of the SNP determination were carried out with different preparations of the PCR templates. To confirm that the PCR products used for the SNP determination were, in fact, *kdr* cDNA fragments, PCR products from one individual of each mosquito samples were sequenced. The alleles at the polymorphism site of each mutation were analyzed using Genemapper software according to the manufacturer’s instructions and as described by Xu et al. [20], [21] and Liu et al. [22]. The frequency (prevalence) of polymorphic expression for each of mutation between and among the groups or strains of the mosquitoes was measured. The statistically significant difference of the frequency of each of the nucleotide polymorphisms between and among the mosquito samples was calculated using a Student's *t*-test for all 2-sample comparisons and a one-way analysis of variance (ANOVA) for multiple sample comparisons (SAS v9.1 software); a value of *P*≤0.05 was considered statistically significant. Pairwise Goeman's Bayesian scores [23] were used for the significant correlation between resistance levels and the SNP combination frequencies of the paired samples using the AssotesteR package in R [24] based on the recommendations for analyzing multiple SNPs in a given gene [25].

## Results

### Isolation of the Full-length Sodium Channel Gene in *Cx. quinquefasciatus*

A 1943 bp partial putative *Culex* sodium channel cDNA fragment was first amplified from S-Lab, HAmCq^G0^, and HAmCq^G8^ by RT-PCR with primers PG KDR S4 and KDR AS02, which were designed from a partial sodium channel cDNA sequence previously generated from *Cx. quinquefasciatus* (Table S2). BLAST analysis showed that this 1943 bp partial putative sodium channel cDNA sequence encoded a putative protein sequences of 511 amino acids and presented at residues 806-1471of the insect sodium channel. We next conducted 5' and 3' RACE to isolate and amplify the full length of the *Culex* sodium channel cDNA using the primer pairs KDR S03/AP1 (the adaptor primer) and KDR AS34/AP1, respectively (Table S2). KDR S03 and KDR AS34 were designed according to the 3' and 5' end sequences of the 1943 bp partial *Culex* sodium channel cDNA fragment. The cDNA sequences amplified by 3' and 5' RACE were overlapped with the corresponding sequences of the 1943 bp partial sodium channel cDNA fragment, revealing them to be the 3' and 5' ends, respectively, of the sodium channel cDNA. The full length of the *Cx. quinquefasciatus* sodium channel cDNA was then isolated for each of S-Lab, HAmCq^G0^, and HAmCq^G8^ mosquitoes (accession numbers: JN695777, JN695778, and JN695779, respectively) by RT-PCR using the primers of KDR S16 and KDR AS09 (Table S2). The full length of the *Cx. quinquefasciatus* sodium channel cDNA contains an open reading frame (ORF) of 6246 nucleotides encoding 2082 amino acids with no frame shifts or premature stop codons present in the sequence (Fig. S1). In this pilot study, the sequence was generated from a pool of mosquito cDNAs for each strain, with a total of 9 complete sodium channel cDNA sequences being analyzed for each.

### Location of Polymorphisms in the Mosquito Sodium Channel

**Figure 1:**
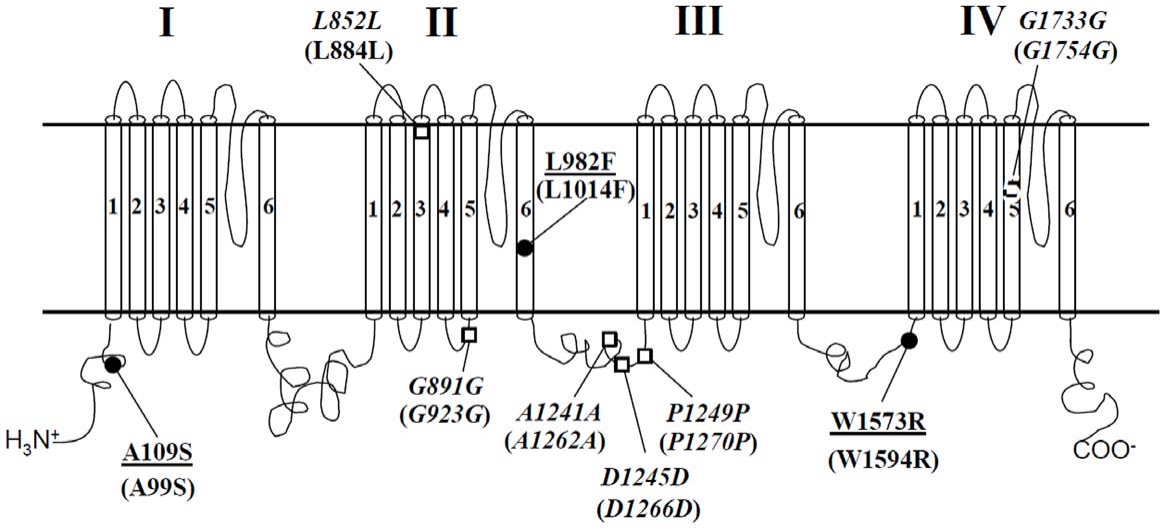
Graphic representation of the locations of synonymous and nonsynonymous mutations in the *Cx. quinquefasciatus* sodium channel. The nonsynonymous mutations are indicated by solid dots and the locations are underlined. The synonymous mutations are indicated by open tetragons and the locations are in italics. Positions of the mutations are numbered according to amino acid sequences of *Cx. quinquefasciatus* (accession numbers: JN695777, JN695778, JN695779), the corresponding positions of which in house fly Vssc1 sodium channel protein are in parentheses. The domain locations of the mutations are assigned according to the sodium channel amino acid sequences in house flies [7], [37].

Comparison of the full length of the *Culex* mosquito sodium channel nucleotide and deduced amino acid sequences was conducted from different mosquito strains, S-Lab, S-Lab^G5^, HAmCq^G0^, and HAmCq^G8^, bearing different resistance phenotypes in response to permethrin (Fig. S1). The criteria for identification were the polymorphisms that were differentially presented among these mosquito strains. This study revealed 3 nonsynonymous mutations, alanine^109^ to serine^109^ (A^109^S), leucine^982^ to phenylalanine^982^ (L^982^F), and tryptophan^1573^ to arginine^1573^ (W^1573^R), resulting from SNPs of guanine to thymine (G325T), adenine to thymine (A2946T), and thymine to cytosine (T4717C), in the sodium channel cDNAs of *Culex* mosquitoes (Fig. S1). In addition, 6 polymorphisms, G2556A, C2673A, A3723G, C3735T, G3747A and A5199G, resulting in synonymous mutations L^852^L, G^891^G, A^1241^A, D^1245^D, P^1249^P, and G^1733^G, were identified (Fig. S1). These 9 mutations scattered along the mosquito sodium channel from N- terminus to the 5^th^ segment of domain IV (IVS5) (Fig. 1).

Among the 3 nonsynonymous mutations, besides the well-known L982F *kdr* mutation in the 6^th^ segment of domain II (IIS6) resistance, two novel mutations, A109S and W1573R, were located at the N- terminus and the intercellular transmembrane linker between IIIS6 and IVS1, respectively, of *Culex* mosquito sodium channel cDNA (Fig. 1). Besides the mutation L^852^ in IIS3 and G^1733^ in IVS5, remaining synonymous mutations were found in the linkers of the sodium channel, with A^1241^, D^1245^, and P^1249^ being located in the intercellular linker between domains II and III and the G^891^ located in the linker connecting IIS4 and IIS5 (Fig. 1).

### Association of Nonsynonymous Mutations with Pyrethroid Resistance in *Cx. quinquefasciatus*

We investigated the evolutionary and heritable adaption of amino acid and codon usage bias, the phenomenon where amino acids or codons are presented with different frequency (prevalence), in mosquitoes following permethrin selection. The SNPs of G325T, A2946T, and T4717C at the codons A^109^S, L^982^F, and W^1573^R, respectively, were examined in adult individuals of each of 4 mosquito strains, S-Lab, S-Lab^G5^, HAmCq^G0^, and HAmCq^G8^. In both the susceptible S-Lab and its permethrin selected offspring S-Lab^G5^ strains, 65 and 70%, respectively, of the tested individuals expressed the susceptible allele G325 at the codon A^109^S, generating a codon encoding alanine; 35 and 30% expressed both the G325 and T325 alleles, and none expressed resistance T325 allele (Table 2). In contrast, 100% tested individuals in the field parental strain (HAmCq^G0^) and their permethrin selected highly resistant offspring (HAmCq^G8^) showed expression of the resistance T325 allele (Table 2), resulting in a substitution of alanine to serine (A^109^S). The fact that the A^109^S occurred in all tested individuals of HAmCq^G0^ strain, coupled with their relatively lower levels of resistance, suggests that this mutation evolved at an early stage of insecticide resistance development and could be responsible for the lower levels of resistance. Alternatively, it may also suggest that the A^109^S is a natural mutation that presented in a highly inbred population but it had nothing to do with resistance.

**Table 2:** Non-synonymous and synonymous mutations in the *Culex* mosquito sodium channel.

Mutation	Strain	n*	Phenotype†	Codons‡ (Frequency [%] ± SE)		
A109S§	S-Lab	60	Susceptible	GCA (65±5.0)	G/TCA(35±5.0)	TCA (0)
	S-Lab^G5^	60	0.9-fold susceptibility	GCA (70±5.0)	G/TCA(30±5.0)	TCA (0)
	HAmCq^G0^	60	10-fold resistance	GCA (0)	G/TCA (0)	TCA (100)
	HAmCq^G8^	60	2,700-fold resistance	GCA (0)	G/TCA (0)	TCA (100)
L982F§	S-Lab	60	Susceptible	TTA (100)	TTA/T (0)	TTT (0)
	S-Lab^G5^	60	0.9-fold susceptibility	TTA (100)	TTA/T (0)	TTT (0)
	HAmCq^G0^	60	10-fold resistance	TTA (20±5.0)	TTA/T (42±3.0)	TTT (38±6.0)
	HAmCq^G8^	60	2,700-fold resistance	TTA (0)	TTA/T (0)	TTT (100)
W1573R§	S-Lab	60	Susceptible	TGG (100)	T/CGG (0)	CGG (0)
	S-Lab^G5^	60	0.9-fold susceptibility	TGG (100)	T/CGG (0)	CGG (0)
	HAmCq^G0^	60	10-fold resistance	TGG (67±11.5)	T/CGG (28±6.0)	CGG (5.0±5.0)
	HAmCq^G8^	60	2,700-fold resistance	TGG (12±7.5)	T/CGG (35±6.0)	CGG (53±3.5)
L852L#	S-Lab	60	Susceptible	CTG (100)	CTG/A (0)	CTA (0)
	S-Lab^G5^	60	0.9-fold susceptibility	CTG (100)	CTG/A (0)	CTA (0)
	HAmCq^G0^	60	10-fold resistance	CTG (52±2.5)	CTG/A (33±7.5)	CTA (15±8.5)
	HAmCq^G8^	60	2,700-fold resistance	CTG (0)	CTG/A (25±5)	CTA (75±5)
G891G#	S-Lab	60	Susceptible	GGC (100)	GGC/A (0)	GGA (0)
	S-Lab^G5^	60	0.9-fold susceptibility	GGC (100)	GGC/A (0)	GGA (0)
	HAmCq^G0^	60	10-fold resistance	GGC (42±5.5)	GGC/A (35±5)	GGA (23±7.5)
	HAmCq^G8^	60	2,700-fold resistance	GGC (0)	GGC/A (8±7.5)	GGA (92±7.5)
A1241A#	S-Lab	60	Susceptible	GCA (100)	GCA/G (0)	GCG (0)
	S-Lab^G5^	60	0.9-fold susceptibility	GCA (100)	GCA/G (0)	GCG (0)
	HAmCq^G0^	60	10-fold resistance	GCA (0)	GCA/G (10±5)	GCG (90±5)
	HAmCq^G8^	60	2,700-fold resistance	GCA (0)	GCA/G (5±0)	GCG (95±0)
D1245D#	S-Lab	60	Susceptible	GAC (100)	GAC/T (0)	GAT (0)
	S-Lab^G5^	60	0.9-fold susceptibility	GAC (100)	GAC/T (0)	GAT (0)
	HAmCq^G0^	60	10-fold resistance	GAC (35±10)	GAC/T (42±2.9)	GAT (23±7.5)
	HAmCq^G8^	60	2,700-fold resistance	GAC (0)	GAC/T (15±5)	GAT (85±5)
P1249P#	S-Lab	60	Susceptible	CCG (100)	CCG/A (0)	CCA (0)
	S-Lab^G5^	60	0.9-fold susceptibility	CCG (100)	CCG/A (0)	CCA (0)
	HAmCq^G0^	60	10-fold resistance	CCG (18±5.5)	CCG/A (53±10)	CCA (29±7.5)
	HAmCq^G8^	60	2,700-fold resistance	CCG (0)	CCG/A (13±7.5)	CCA (87±7.5)
G1733G#	S-Lab	60	Susceptible	GGA (48±12.5)	GGA/G (52±12.5)	GGG (0)
	S-Lab	60	0.9-fold susceptibility	GGA (55±7.5)	GGA/G (45±10)	GGG (0)
	HAmCq^G0^	60	10-fold resistance	GGA (0)	GGA/G (3±2.9)	GGG (97±2.9)
	HAmCq^G8^	60	2,700-fold resistance	GGA (0)	GGA/G (0)	GGG (100)

G0 represents the parental insects collected directly from the field; G8 represent the 8^th^ generation of permethrin-selected HAmCq^G0^ offspring; Values represent mean ± SE for three replications of frequency (%) analyses of each mutation.

* The total number of tested adult mosquitoes (three replicates for each of 20 [10 males and 10 females]).

† Data from [18] except that for S-Lab^G5^, which was tested in the current study (Table S2).

‡ The nucleotide polymorphisms are underlined.

§ Non-synonymous mutations.

# Synonymous mutations.

A strong correlation between the prevalence of polymorphic expression of A2946T and T4717C at the codons L^982^F and W^1573^R, respectively, and the levels of pyrethroid resistance in *Culex* mosquitoes was identified. All tested individuals in the susceptible S-Lab and S-Lab^G5^ strains expressed the susceptible polymorphisms A2946 and T4717, respectively, producing codons encoding leucine (L^982^) and tryptophan (W^1573^) (Table 2). In contrast, all tested individuals in HAmCq^G8^ expressed polymorphic allele T2946, producing a substitution codon encoding phenylalanine (F^982^) and the majority of this strain expressed polymorphic allele C4717, generating a substitute codon encoding arginine (R^1573^), corresponding to their high levels of resistance. Whereas, HAmCq^G0^ showed an intermediate level of allelic expression for SNPs of T2946A and T4717C, corresponding to their low level of resistance (Table 2). Unlike the field mosquito strain HAmCq^G0^, which showed the increased levels of resistance to permethrin and the frequency of polymorphic expression of sodium channel mutations after selection (HAmCq^G8^), the permethrin selected S-Lab strain, S-Lab^G5^, showed no change in the susceptibility to peremthrin and the frequency of polymorphic expression compared with S-Lab. These results suggest not only that the L^982^F and W^1573^R mutations are highly likely to be involved in the mosquitoes’ elevated levels of pyrethroid resistance, revealing the inheritance and evolution of the codon usage bias in the field mosquito strain to be controlled by permethrin selection but also that no individuals with polymorphic alleles are presented in the laboratory susceptible S-Lab strain.

### Six Synonymous Mutations Associated with Pyrethroid Resistant Mosquitoes

The SNP determination also revealed strong correlations between the frequency of polymorphic expression at the 6 synonymous codon sites and the levels of susceptibility and resistance in *Cx. quinquefasciatus* (Table 2). All the synonymous nucleotide polymorphisms, same as those nonsynonymous polymorphisms, showed a strong association between the prevalence of polymorphic codon usage and the evolution of permethrin selection (Table 2). Non nucleotide substitutions at the synonymous codon sites, besides G^1733^G, were detected in both S-lab and S-Lab^G5^ mosquito strains; higher frequencies of the polymorphic expression were detected in HAmCq^G8^; and relatively low frequencies were detected in HAmCq^G0^ (Table 2). Only the polymorphism of A5199G at the codon G^1733^G showed a relative high frequency (97%) of the polymorphic expression in HAmCq^G0^ (Table 2), suggesting that synonymous polymorphism A5199G at the codon G^1733^G may, as with the nonsynonymous A^109^S mutation, evolve in the earliest stage of permethrin selection and is thus important for the development of low levels of pyrethroid resistance.

### Distribution of Polymorphic Allele Frequencies and their Correlation with the Tolerance of Mosquitoes to Permethrin

**Figure 2:**
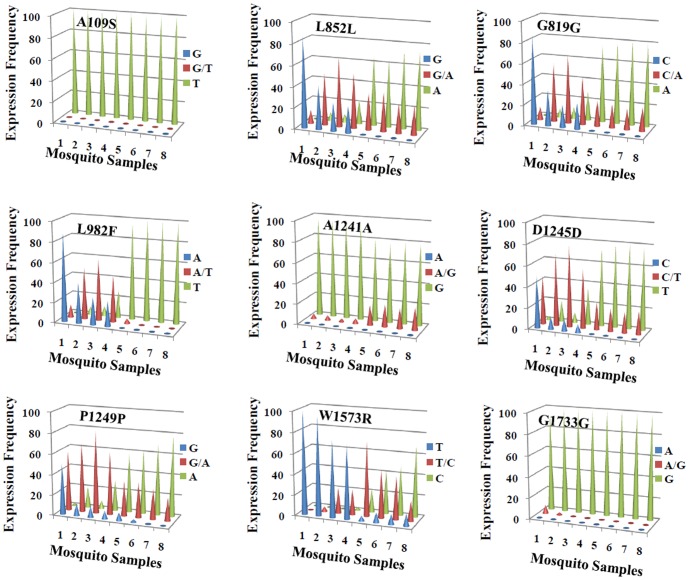
Distribution of frequencies of allelic expression in mosquito groups threated with permethrin. The frequency of allele expression shown along the Y axis is the percentage of the mosquitoes (n = 40) carrying the homozygous or heterozygous allele(s) of the mutation. Mosquito groups are shown along the X axis; 1, 2, 3, and 4 represent the groups in HAmCq^G0^ that are dead under LC_10_ concentration treatment, between LC_10_ and LC_50_, between LC_50_ and LC_90_ and alive above LC_90_, respectively; and 5, 6, 7, and 8 represent the groups in HAmCq^G8^ that are dead under LC_10_, between LC_10_ and LC_50_, between LC_50_ and LC_90_, and alive above LC_90_, respectively.

To further evaluate the role of the polymorphisms in pyrethroid resistance of mosquitoes, the prevalence of each sodium channel mutation correlated with the mosquitoes’ tolerance to certain concentrations of permethrin was examined in HAmCq^G0^ and its permethrin selected offspring HAmCq^G8^. We treated mosquito larvae of each strain with different concentration of permethrin (Table 1) and assembled them into four groups (1 to 4) based on their similar levels of tolerance to permethrin (low to high). The results showed that all or most of the individuals in all tested groups across the field parental and permethrin selected offspring strains were homozygous for polymorphic alleles T325 and G5199 at codons A^109^S and G^1733^G, respectively (Table 3). This result was strongly consistent with our findings from the association study of mutations with pyrethroid resistance with adult mosquitoes (Table 2), strengthening our earlier suggestion that mutations at codons A^109^S and G^1733^G may evolve in the earliest stage of permethrin resistance and are thus important for the development of low levels of pyrethroid resistance. Nevertheless, a significantly different distribution of the frequency of polymorphisms for the remainder of the 7 nonsynonymous and synonymous mutations was found among different groups of mosquito strains (Fig. 2). The individuals carrying homozygous susceptible alleles gradually decreased and the individuals carrying homozygous polymorphic alleles gradually increased following the increased tolerance of the groups to permethrin treatments. Individuals carrying heterozygous mutations showed a strong transitional phase from lower tolerance to high. The correlation of the mutation prevalence with the level of tolerance to permethrin revealed that these 7 mutations were highly likely to be directly relevant to resistance evolution and inheritance of mutations through the generations of permethrin selection. The homozygous polymorphic alleles A2556, A2673, T2946, G3723, T3735, and A3747 appeared starting from group 2 of HAmCq^G0^, with a tolerance to permethrin concentrations between 0.005 and 0.05 ppm (Table 1), falling concentration range of LC_10_ to LC_50_. This result suggests that homozygous A2556, A2673, T2946, G3723, T3735, or A3747 polymorphisms may responsible for the initiation of moderate levels of permethrin resistance. Mutation C4717 emerged starting from group 4 of HAmCq^G0^, which exhibited tolerance to permethrin concentration of more than LC_90_ (>0.2 ppm).

**Table 3:** Co-occurrence of the *kdr* mutations in the HAmCq groups with difference levels of tolerance to permethrin.

				Polymorphisms at Amino Acid Mutation Sites								
				A109S	L852L	G891G	L982F	A1241A	D1245D	P1249P	W1573R	G1733G
*Mosquito Groups		†N	‡F% (SE)	G to T	G to A	C to A	A to T	A to G	C to T	G to A	T to C	A to G
HAmCqG^0^	1	1	7.5 (3.5)	T	G	C	A	G	C	G	T	A/G
		2	42.5 (10)	T	G	C	A	G	C	G	T	G
		3	5 (0)	T	G	C	A	A/G	C/T	G/A	T	G
		4	32.5 (3.5)	T	G	C	A	G	C/T	G/A	T	G
		5	10 (0)	T	G/A	C/A	A/T	A/G	C/T	G/A	T	G
		11	2.5 (3.5)	T	G/A	C/A	A/T	G	T	A	T	G
	2	1	2.5 (3.5)	T	G	C	A	G	C	G	T	A/G
		2	10 (7)	T	G	C	A	G	C	G	T	G
		4	27.5 (3.5)	T	G	C	A	G	C/T	G/A	T	G
		5	5 (0)	T	G/A	C/A	A/T	A/G	C/T	G/A	T	G
		8	32.5 (3.5)	T	G/A	C/A	A/T	G	C/T	G/A	T	G
		9	2.5 (3.5)	T	G/A	C/A	A/T	G	C/T	G/A	T/C	G
		11	12.5 (3.5)	T	G/A	C/A	A/T	G	T	A	T	G
		17	5 (0)	T	A	A	T	G	T	A	T	G
		19	2.5 (3.5)	T	A	A	T	G	T	A	T/C	G
	3	2	12.5 (3.5)	T	G	C	A	G	C	G	T	G
		4	7.5 (3.5)	T	G	C	A	G	C/T	G/A	T	G
		5	2.5 (3.5)	T	G/A	C/A	A/T	A/G	C/T	G/A	T	G
		6	7.5 (3.5)	T	G	C	A	G	C/T	G/A	T/C	G
		8	47.5 (10.5)	T	G/A	C/A	A/T	G	C/T	G/A	T	G
		9	12.5 (3.5)	T	G/A	C/A	A/T	G	C/T	G/A	T/C	G
		12	2.5 (3.5)	T	G/A	A	T	G	T	G/A	T	G
		17	3.5 (3.5)	T	A	A	T	G	T	A	T	G
		19	5 (0)	T	A	A	T	G	T	A	T/C	G
	4	2	7.5 (3.5)	T	G	C	A	G	C	G	T	G
		4	12.5 (3.5)	T	G	C	A	G	C/T	G/A	T	G
		5	5 (0)	T	G/A	C/A	A/T	A/G	C/T	G/A	T	G
		6	5 (0)	T	G	C	A	G	C/T	G/A	T/C	G
		8	27.5 (10.5)	T	G/A	C/A	A/T	G	C/T	G/A	T	G
		9	7.5 (3.5)	T	G/A	C/A	A/T	G	C/T	G/A	T/C	G
		11	7.5 (3.5)	T	G/A	C/A	A/T	G	T	A	T	G
		12	5 (0)	T	G/A	A	T	G	T	G/A	T	G
		17	7.5 (3.5)	T	A	A	T	G	T	A	T	G
		19	12.5 (3.5)	T	A	A	T	G	T	A	T/C	G
		20	2.5 (3.5)	T	A	A	T	G	T	A	C	G
HAmCqG^10^	1	7	2.5 (3.5)	T	G/A	C/A	T	A/G	C/T	G/A	T	G
		9	5 (0)	T	G/A	C/A	A/T	G	C/T	G/A	T/C	G
		10	17.5 (3.5)	T	G/A	C/A	T	A/G	C/T	G/A	T/C	G
		14	10 (0)	T	A	A	T	G	T	G	C	G
		15	7.5 (3.5)	T	A	A	T	G	T	G/A	T/C	G
		16	10 (7)	T	G/A	A	T	G	T	A	T/C	G
		19	35 (7)	T	A	A	T	G	T	A	T/C	G
		20	12.5 (3.5)	T	A	A	T	G	T	A	C	G
	2	7	10 (7)	T	G/A	C/A	T	A/G	C/T	G/A	T	G
		10	12.5 (3.5)	T	G/A	C/A	T	A/G	C/T	G/A	T/C	G
		13	7.5 (3.5)	T	G/A	A	T	G	T	G/A	T/C	G
		14	2.5 (3.5)	T	A	A	T	G	T	G	C	G
		15	5 (0)	T	A	A	T	G	T	G/A	T/C	G
		18	7.5 (3.5)	T	G/A	A	T	G	T	A	C	G
		19	22.5 (10.5)	T	A	A	T	G	T	A	T/C	G
		20	32.5 (3.5)	T	A	A	T	G	T	A	C	G
	3	7	10 (7)	T	G/A	C/A	T	A/G	C/T	G/A	T	G
		10	10 (0)	T	G/A	C/A	T	A/G	C/T	G/A	T/C	G
		15	10 (7)	T	A	A	T	G	T	G/A	T/C	G
		18	7.5 (3,5)	T	G/A	A	T	G	T	A	C	G
		19	22.5 (10.5)	T	A	A	T	G	T	A	T/C	G
		20	40 (7)	T	A	A	T	G	T	A	C	G
	4	7	12.5 (3.5)	T	G/A	C/A	T	A/G	C/T	G/A	T	G
		10	10 (7)	T	G/A	C/A	T	A/G	C/T	G/A	T/C	G
		18	5 (0)	T	G/A	A	T	G	T	A	C	G
		19	10 (0)	T	A	A	T	G	T	A	T/C	G
		20	62.5 (10.5)	T	A	A	T	G	T	A	C	G

* Group 1 were mosquitoes with tolerance to the permethrin concentration <LC_10_ (i.e., HAmCq^G0^-<LC_10_, and HAmCq^G8^-<LC_10_); group 2 were the mosquitoes with the tolerance to permethrin between LC_10_ - LC_50_ concentrations (i.e., HAmCq^G0^-LC_10–50_, and HAmCq^G8^-LC_10–50_); group 3 were the mosquitoes with the tolerance to permethrin between LC_50_ - LC_90_ concentrations (i.e., HAmCq^G0^-LC_50–90_, and HAmCq^G8^-LC_50–90_); and group 4 were the mosquitoes with the tolerance to the permethrin concentration >LC_90_ (i.e., HAmCqG0->LC_90_, and HAmCq^G8^->LC_90_) (Table 1)_._

† N: The numeral indicating each different combination of the mutations, which was designed by weighing the numbers of the homozygous susceptible alleles, heterozygous, and homozygous polymorphic alleles in the combination, i.e., the numeral was increased when the heterozygous and homozygous polymorphic alleles increased.

‡ F: the frequency (%) of each of the mutation combinations occurred in each group. The total of 40 individuals (two replicates for each of 20 4^th^ instar larvae) with all ten mutations in their sodium channel cDNAs was analyzed.

### Collaborative Effects of the Multiple Mutations of the Sodium Channel in Mosquitoes’ Response to Permethrin

To investigate the effects of different mutation combinations in mosquito resistance and the specific thresholds of insecticide concentrations, at which particular mutations or mutation combinations occurs in a mosquito strain, we examined the frequency of particular synonymous and/or nonsynonymous mutations that co-occur in the mosquito groups across different strains. Applying critical criterion specifying that only individuals in which all 9 mutations could be detected in their full length sodium channel would be utilized for the data analyses, sodium channel mutations were analyzed in a total of 40 individuals in each of the mosquito groups. A total of 20 mutation combinations were identified across the mosquito strains and groups (Table 3). Our results revealed a general and clear shift of mutation combinations from those with most homozygous susceptible alleles through those with the most intermediate heterozygous alleles to those with the most or all homozygous polymorphic alleles at the mutation sites corresponding to the increasing tolerance of the mosquito groups to permethrin treatments in both mosquito strains. The most dominant mutation combination(s) observed in each of groups and strains were identified (Table 3). The category #8 (triple homozygous mutations and quintuple heterozygous mutations; T^325^, g/a^2556^, c/a^2673^, a/t^2946^, G^3723^, c/t^2735^, g/a^3747^, G^5199^) was the predominant mutation combination in group 2 of the field parental mosquito strain of HAmCq^G0^, with prevalence of 32.5. Category #8 was also the dominant mutation combination in groups 3 and 4 (with frequencies of 47.5 and 27.5%, respectively) of HAmCq^G0^. These results strongly revealed the importance of this mutation combination (#8) in the development of insecticide resistance in field strains. The first occurrence of the category #8 combination was in the group 2 mosquitoes of HAmCq^G0^, which had a tolerance to permethrin concentrations between 0.005 and 0.05 ppm (LC_10_ to LC_50,_ Table 1), suggesting that the concentration range of 0.005 to 0.05 ppm represents a threshold at which the #8 mutation occurs in a mosquito strain. The occurrence of category #20 (nonuple homozygous mutations, T^325^, A^2556^, A^2673^, T^2946^, G^3723^, T^2735^, A^3747^, C^4717^ and G^5199^) emerged in the group 4 mosquitoes of HAmCq^G0^ with very low frequencies of 2.5, suggesting that permethrin concentrations at or >0.2 ppm may represent the threshold at which the particular #20 mutation combination occurs in field mosquito strains.

Comparing mutation combinations in permethrin selected offspring HAmCq^G8^ with their field parental mosquitoes HAmCq^G0^ revealed a clear shift in the mutation combinations in these strains from the majority being heterozygous mutation combinations, for example categories #8 in HAmCq^G0^, to the majority being resistance homozygous combinations such as category #20 in HAmCq^G8^ (Table 3). Pairwise Goeman's Bayesian scores (Goeman et al., 2006) tested with the software of AssotesteR package in R (Sanchez, 2012) revealed the significant correlation between resistance levels of mosquito groups and the SNP combination frequencies of them (Table 4). A significant (P≤0.05) transition in the prevalence of the nonuple homozygous mutation combinations (category #20) was observed between the field parental strain and its permethrin selected offspring (Table 3), revealing a clear-cut pattern of inheritance of the mutations. Although category #20 was the major mutation combination in all 4 groups of HAmCq^G8^, a significant shift in prevalence of this mutation combination was also observed from 12.5% in group 1 with the lowest level of tolerance to permethrin treatment to 62.5% in group 4 with the highest level of tolerance to permethrin treatment in HAmCq^G8^. The strong correlation of the frequency of the mutation combination and association with permethrin selection and tolerance to permethrin treatment confirmed that not only are these mutations co-selected by permethrin, but the combination of all 9 mutations is also involved in the high levels of resistance.

**Table 4:** Pairwise Goeman's Bayesian score test values to check for correlation between SNP combination frequencies and permethrin resistance level.

		HAmCq^G0^				HAmCq^G8^			
Strain	Group	1	2	3	4	1	2	3	4
HAmCq^G0^	1	–							
	2	290***	–						
	3	360***	7.7	–					
	4	670***	69**	35*	–				
HAmCq^G8^	1	2400***	1100***	90***	560***	–			
	2	2500***	1200***	1000***	650***	−7.5	–		
	3	2700***	1300***	1100***	730***	4.7	−9.1	–	
	4	2800***	1400***	1200***	770***	30*	3.7	−6.1	–

* *P<*0.1;

** *P*<0.05;

*** *P*<0.001.

† Goeman’s Bayesian score test value based on 500 permutations. The Goeman’s Bayesian score represent a relative value of comparison of the paired samples. The higher the score values the more significant the correlation between resistance level and the SNP combination frequencies of paired samples.

## Discussion

Insecticide resistance is generally assumed to be a pre-adaptive phenomenon, in which prior to insecticide exposure rare individuals carrying an altered (varied) genome already exist, allowing survival of those carrying the genetic variance from insecticide selection [26]. Accordingly, the individuals carrying the resistance genes, polymorphisms or alleles should increase in a strain following selection through the inheritance and become predominate in the strain. Our approach of comparing the polymorphic codon usage bias along the full length of the sodium channel cDNA sequences in individuals among different mosquito strains and groups and between parental field strains and their permethrin selected offspring with different levels of insecticide resistance allowed us to not only examine the polymorphisms themselves but also to evaluate their evolutionary and heritable feature following permethrin selection. Furthermore, the analysis of *kdr* mutations over the entire mosquito sodium channel, along with an examination of the mutation combinations in different mosquito groups categorized by their levels of tolerance to a range of permethrin concentrations within and among the field parental strain and their permethrin selected offspring, for the first time, allowed us to characterize the specific thresholds of insecticide concentrations at which particular mutations or mutation combinations occur in a mosquito strain. Adopting this strategy enabled us to identify a total of 9 mutations, 3 nonsynonymous and 6 synonymous, in the *Culex* mosquito sodium channel of individual resistant mosquitoes. Beside the well-known L-to-F mutation in IIS6, the other 2 nonsynonymous mutations, were located at the N- terminus and the intercellular transmembrane linker between IIIS6 and IVS1, in which several nonsynonymous mutations, R52Q, D59G, L1770P, D1561V, and E1565G, have been identified in pyrethroid resistant insects and mites [4], [27]–[31]. It has also been demonstrated that the linker region between III and IV is functionally important in channel inactivation [32]–[34]. Among 6 synonymous mutations, 4 of them were found in the intercellular linkers between domains II and III and between IIS4 and IIS5.

Although to date no synonymous mutations have been intensely characterized in insect sodium channels, several nonsynonymous mutations located in the linker regions connecting the sodium channel have been identified in pyrethroid resistant insects and arthropods. An A1060T nonsynonymous mutation has been identified in the intercellular linker between domains II and III of the sodium channel in pyrethroid resistant diamondback moths, *Plutella xylostella* [35]. A nonsynonymous substitution A1215D that lies within the same intracellular linker has also been proposed as a likely contributor to the resistance phenotype in the two-spotted spider mite *Tetranychus urticae* [36]. A nonsynonymous mutation M918T, termed a *super*-*kdr* mutation, has been identified in the linker connecting IIS4 and IIS5 in house flies, *Musca domestica* [7], [8], [37] and horn flies, *Hematobia irritans* [38]. Two nonsynonymous mutations, M918V and L925I, have been identified in the linker connecting IIS4 and IIS5 of sodium channels in whiteflies, *Bemisia tabaci* [39]. Nevertheless, to date nor substitutions in IIS3 and IVS5 have been reported. Yet, although several synonymous mutations in insect sodium channel genes have been identified by previous researchers [37], [40], their role in insecticide resistance has not been explored. Recent research suggests that synonymous mutations may be associated with a variety of biological factors and may play a significant role in altering gene functions, including gene expression [41], formation of secondary structures of proteins [42], protein folding and substrate/protein interaction [43]. Thus, there is a reason to re-consider the important role that synonymous mutations identified in the sodium channel of the insect nervous system may play in insecticide resistance. We identified 6 synonymous mutations present in the *Culex* mosquito sodium channel, 5 of which evolved following permethrin selection, suggesting an important role of these mutations in the evolution of permethrin selection and the development of insecticide resistance. Whether these nonsynonymous mutations in the mosquito sodium channel gene perform similar functions to those described above, particularly the question of whether they are involved in altering the structure of the permethrin interaction sites in the sodium channel requires further investigation.

The synergistic effects of co-existence of insect sodium channel mutations on insecticide resistance have been reported. The most notable one is the co-presence of the methionine (M) to threonine (T) mutation (M918T), termed a *super*-*kdr* mutation in the linker connecting IIS4 and IIS5, with the L to F (L1014F) mutation in IIS6 of the sodium channel in super-kdr house flies, which exhibit higher levels of resistance to DDT and pyrethroids than kdr house flies, where only the L1014F mutation is observed [3], [4], [7], [8]. Besides co-existing of L1014F and M918T mutation in the super-kdr house flies, the same combination of M-to-T and L-to-F mutations corresponding to house fly L1014F and M918T is also found in other insect species, such as *Haematobia irritans* [44], *Thrips tabaci* [45], and *Myzus persicae* [46], all of which have been found to exhibit relatively high-level resistance to pyrethroids. The *super kdr* mutation has not been reported in other insect species apart from those mentioned above, including mosquitoes. It has, however, been reported that, instead of the M-to-T *super kdr* mutation, additional sodium channel mutations may co-exist with the L-to-F mutation that are associated with high levels of resistance [3]–[5]. In contrast, some *kdr*-type insect strains lack the L-to-F/H/S mutation and other co-existing sodium channel mutations are often found in these resistant insects [47].

Our study, for the first time, evaluated mutation combinations over the entire sodium channel of individual mosquitoes in a resistant field strain, the laboratory selected offspring of these field strains, and groups of mosquitoes with different levels of tolerance to permethrin treatment within and among the mosquito strains. Our data provide a strong case demonstrating the co-existence of both nonsynonymous and synonymous mutations in the sodium channel of individual resistant mosquitoes in response to insecticide resistance. Although M to T *super kdr* mutation in the linker connecting IIS4 and IIS5 has was not identified in the sodium channel sequences of any individual mosquitoes that we tested, the synonymous polymorphism of cytosine to adenine (C2673A) at the codon G^891^G (corresponding to G^923^ of the house fly Vssc1 sodium channel protein) was found in all field parental and permethrin selected mosquito individuals tested. The synonymous codon G^891^G is located in the linker connecting IIS4 and IIS5, five amino acids downstream from the methionine residue (corresponding to the position of the M918T mutation in the house fly Vssc1 sodium channel protein [7], [37]. Our results also showed that not only the C2673A synonymous polymorphism was almost always linked with the L^982^F mutation (corresponding to the position of the L1014F mutation in house fly Vssc1) in resistant *Culex* mosquitoes but also that they co-presented together with other mutations in resistant mosquitoes. Synergistic effects on resistance were detected in all resistant mosquito strains tested following the increasing number of mutations and their homozygosity.

Analyses of *kdr* mutation combinations in different mosquito groups categorized by their levels of tolerance to a range of permethrin concentrations, for the first time, allowed us to characterize specific thresholds of insecticide concentrations at which particular mutations or mutation combinations occur in a mosquito strain. These findings provide important information not only for diagnosis of sodium channel mutations-mediated insecticide resistance in field mosquito strains but also for estimation of the levels of resistance by analyzing the mutation combinations. Yet, future research should focus on investigating the function and interaction of these mutations and how it affects the structure and function of sodium channel proteins, particularly with regard to the gating properties of the sodium channel and the binding configurations of the sodium channel to insecticides. The roles of synonymous mutations in sodium channel functions should also be examined in terms of protein secondary structure formation [42] and protein folding [43], as this should shed new light on how the molecular mechanisms of sodium channel insensitivity act to mediate insecticide resistance.

The accession numbers for the sodium channel sequences of S- Lab, HAmCq^G0^ and HAmCq^G8^ are JN695777, JN695778, and JN695779, respectively.

## Supporting Information

Figure S1 **Alignment of the cDNA/deduced amino acid sequences in mosquitoes.** The cDNA/deduced amino acid sequences of the full length *Cx. quinquefasciatus* sodium channel cDNA were compared among S-Lab, HAmCq^G0^, and HAmCq^G8^ (Accession numbers: JN695777, JN695778, JN695779). The nucleotides/deduced amino acids are numbered from transcription start point (*tsp*)/translation start codon (*tsc*) as +1. The *tsp*/*tsc* and a polyadenylation signal are in bold and italics. The nonsynonymous codons and corresponding amino acid substitutions are highlighted and polymorphism codes are underlined. The synonymous codons are highlighted and polymorphism codes are doubly underlined. In this pilot study, the sequence was generated from a pool of mosquito cDNAs for each of three mosquito strains, with a total of 9 complete sodium channel cDNA sequences being analyzed for each. Unlike the other nonsynonymous and synonymous mutations, SNPs of which were only presented in the resistant HAmCq^G0^ and HAmCq^G8^ strains, both homozygous susceptible alleles and heterozygous alleles at the codons of the nonsynonymous A^109^S and synonymous G^1752^G were presented in the susceptible S-lab mosquitoes, only the homozygous polymorphic alleles was observed in the highly resistant HAmCq^G8^ strain.
(DOC)

Table S1 **Toxicity of permethrin to the S-Lab strain before and after permethrin selection.** ^a^Number of selected fourth instar larvae ^b^LC_50_ values in ppm ^c^95% confidence interval, toxicity of permethrin is considered significantly different when the 95% CI fail to overlap ^d^RR: LC_50_ of selected generations/LC_50_ of the S-Lab strain ^e^Parental strain – i.e., S-Lab
(DOC)

Table S2 Oligonucleotide primers^*^ used for amplifying the sodium channel cDNA, qRT-PCR reactions and SNP (single nucleotide polymorphism) determination. ^*^Designation of oligonucleotide mixtures: R = A+G; Y = C+T; K = G+T.
(DOC)
