# Supplementary material for: Evolutionary Adaptation of the Amino Acid and Codon Usage of the Mosquito Sodium Channel following Insecticide Selection in the Field Mosquitoes
Source: PLoS One. 2012 Oct 17;7(10):e47609. doi: 10.1371/journal.pone.0047609 (PMC3474719; doi:10.1371/journal.pone.0047609)
Supplement: Figure S1 — Alignment of the cDNA/deduced amino acid sequences in mosquitoes. The cDNA/deduced amino acid sequences of the full length Cx. quinquefasciatus sodium channel cDNA were compared among S-Lab, HAmCqG0, and HAmCqG8 (Accession numbers: JN695777, JN695778, JN695779). The nucleotides/deduced amino acids are numbered from transcription start point (tsp)/translation start codon (tsc) as +1. The tsp/tsc and a polyadenylation signal are in bold and italics. The nonsynonymous codons and corresponding amino acid substitutions are highlighted and polymorphism codes are underlined. The synonymous codons are highlighted and polymorphism codes are doubly underlined. In this pilot study, the sequence was generated from a pool of mosquito cDNAs for each of three mosquito strains, with a total of 9 complete sodium channel cDNA sequences being analyzed for each. Unlike the other nonsynonymous and synonymous mutations, SNPs of which were only presented in the resistant HAmCqG0 and HAmCqG8 strains, both homozygous susceptible alleles and heterozygous alleles at the codons of the nonsynonymous A109S and synonymous G1752G were presented in the susceptible S-lab mosquitoes, only the homozygous polymorphic alleles was observed in the highly resistant HAmCqG8 strain. (DOC) [file pone.0047609.s001.doc]

***ATG***ACCGAAGACCTCGATTCGATATCTGAGGAAGAACGTAGTTTGTTCCGTCCTTTCACC 60

***M*** T E D L D S I S E E E R S L F R P F T 20

CGTGAATCATTACTTGTTATTGAAGAACGCATCGCAAATGAACAGGCAAAACAGCGCGAA 120

R E S L L V I E E R I A N E Q A K Q R E 40

TTGGAAAAGAAGCGAGCCGAGGGGGAGACTGGTTTTGGTCGGAAGAAAAAGAAAA**A**AGAA 180

L E K K R A E G E T G F G R K K K K **K** E 60

ATACGGTACGACGATGAGGACGAGGATGAAGGTCCC**C**AGC**C**GGACTCCACACTCGAGCAG 240

I R Y D D E D E D E G P **Q** **P** D S T L E Q 80

GGAGTGCCGATCCCTGTTCGAATGCAGGGCAGCTTCCCTCCGGAATTGGCCTCCACGCCT 300

G V P I P V R M Q G S F P P E L A S T P 100

CTCGAGGATATTGACGCTTTTTAC**G**CAAACATTAAGACATTCGTAGTAGTTAGTAAAGGA 360

------------------------**T**-----------------------------------

L E D I D A F Y **A** N I K T F V V V S K G 120

- - - - - - - - **S** - - - - - - - - - - -

AAGGATATTTTTCGTTTCTCCGCAACCAATGCATTATATGTACTCGATCCGTTCAATCCT 420

K D I F R F S A T N A L Y V L D P F N P 140

ATACGTCGCGTAGCTATTTATATTTTAGTACATCCATTATTTTCATTTTTTATAATAACA 480

I R R V A I Y I L V H P L F S F F I I T 160

ACCATTCTTGGTAATTGTATTTTGATGATCATGCCATCCACGCCGACAGTCGAATCTACC 540

T I L G N C I L M I M P S T P T V E S T 180

GAGGTGATATTCACCGGCATCTACACGTTCGAATCAGCTGTAAAAGTGATGGCGCGAGGT 600

E V I F T G I Y T F E S A V K V M A R G 200

TTCATATTACAACCGTTTACTTATCTTAGAGATGCATGGAATTGGTTGGACTTCGTAGTA 660

F I L Q P F T Y L R D A W N W L D F V V 220

ATAGCATTAGCATATGTAACTATGGGTATAGATTTGGGTAATCTCGCTGCATTGAGAACA 720

I A L A Y V T M G I D L G N L A A L R T 240

TTCAGGGTACTACGAGCTC**C**CAAAACAGTGGCCATCGTTCCAGGTCTCAAGACCATCGTC 780

F R V L R A **P** K T V A I V P G L K T I V 260

GGCGCTGTCA**T**CGAGTCCGTAAAGAATCTCAGAGATGTGATAATTTTAACAATGTTTTCG 840

G A V **I** E S V K N L R D V I I L T M F S 280

TTGTCGGTGTTTGCTTTAATGGGGCTGCAGATCTACATGGGCGTGCTGACGCAAAAGTGC 900

L S V F A L M G L Q I Y M G V L T Q K C 300

ATCAAGGAGTTCCCGACGGACGGCTCGTGGGGCAACCTGACCCACGAGAACTGGGAGCGG 960

I K E F P T D G S W G N L T H E N W E R 320

CACCATTCGAACGATTCCAATTGGTACTTTTCCGAAACCGGGGACACGCCCCTCTGCGGC 1020

H H S N D S N W Y F S E T G D T P L C G 340

AATTCGTCGGGTGCTGGCCAATGTGAGGAAGGATATGTATGTTTACAAGGTTTTGGAGAT 1080

N S S G A G Q C E E G Y V C L Q G F G D 360

AATCCA**A**ATTACGGGTATACAAGTTTTGATACTTTCGGATGGGCATTCTTATCTGCCTTT 1140

N P **N** Y G Y T S F D T F G W A F L S A F 380

CGTCTCATGACCCAGGACTACTGGGAGAATTTATATCAACTGGTGTTACGATCAGCTGGA 1200

R L M T Q D Y W E N L Y Q L V L R S A G 400

CCGTGGCACATGCTCTTCTTCATTGTGATTATCTTCT**T**GGGTTCGTTCTACCTTGTA**A**AT 1260

P W H M L F F I V I I F **L** G S F Y L V **N** 420

TTGATCTTGGCCATTGTCGCCATGTCGTACGACGAACTCCAGAAGAGGGCCGAAGAGGAA 1320

L I L A I V A M S Y D E L Q K R A E E E 440

GAGGCCGCCGAGGAAGAAGCGCTTCGGGAAGCGGAAGAAGCGGCCGCAGCGAAACAGGCC 1380

E A A E E E A L R E A E E A A A A K Q A 460

A**A**ACTCGAGGCCCACGCAGCGGCGGCGGCGGCCGCAGCCAACCCGGAGATCGCCAAGAGC 1440

**K** L E A H A A A A A A A A N P E I A K S 480

CCGTCGGACTTTTCCTGCCACAGTT**G**CGAGCTGTTCGTGGGCCAGGAGAAGGGCAACGAC 1500

P S D F S C H S **C** E L F V G Q E K G N D 500

GACAACAACA**AG**GAGAAGATGTCGATCCGGAGCGAAGGATTGGAGTCGGCTTCACTTTCA 1560

D N N **K** E K M S I R S E G L E S A S L S 520

TTACCTGGTTCACCAT**T**TAATCTTCGTAGAGGATCTAGAGGATCACATCAGTTTACGATA 1620

L P G S P **F** N L R R G S R G S H Q F T I 540

CGTAACGGTAGAGGACGTTTCGTGGGCGTACCTGGTAGCGATAGAAAACCATTGGTACTC 1680

R N G R G R F V G V P G S D R K P L V L 560

TCAACATATCTCGATGCACAAGAACACTTGCCATACGCCGATGACTCGAACGCGGTCACA 1740

S T Y L D A Q E H L P Y A D D S N A V T 580

CCGATGTCGGAGGAAAATGGTTCGCGACACTCATCGTACACATCGCATCAATCGCGCATC 1800

P M S E E N G S R H S S Y T S H Q S R I 600

TCGTACACATCGCACGGCGACCTGCTCGGCGGCATGACGAAGGAGAGCCGGCTGCGGAGC 1860

S Y T S H G D L L G G M T K E S R L R S 620

CGAACCCAGCGCAACACGAACCACTCGATCGTGCCGCCGGCGAACATGGCGGCCTCGGCG 1920

R T Q R N T N H S I V P P A N M A A S A 640

GCGTCGGTGACGGGTGCCGGCTCGGGCGCGCCCAACATG**C**CCTACGTCGACACCAACC**C**C 1980

A S V T G A G S G A P N M **P** Y V D T N **P** 660

AAGGGCCAGCAGCGCGACTTTGATCAGTCCCAAGACTACACAGATGATGCTGGTAAAATA 2040

K G Q Q R D F D Q S Q D Y T D D A G K I 680

AAACACAACGACAATCCTTTCATCGAGCCCTCTCAAACCCAAACCGTAGTAGATATGAAA 2100

K H N D N P F I E P S Q T Q T V V D M K 700

GACGTAATGGTGTTAAACGATATCATTGAGCAAGCTGCTGGTCGGCATAGTAGAGCTAGT 2160

D V M V L N D I I E Q A A G R H S R A S 720

GATCATGGAGAGGACGACGACGAGGACGGTCCGACG**T**TCAAG**C**ACAAGGCGG**C**CGAGTTC 2220

D H G E D D D E D G P T **F** K **H** K A **A** E F 740

GGGATGCGGATGATCGACATCTTCTGCGTGTGGGACTGCTGCTGGGTGTGGCTCAAGTTC 2280

G M R M I D I F C V W D C C W V W L K F 760

CAGGAGTGGG**T**GTCCTTTATCGTGTTCGACCCGTTCGTCGAGCTGTTCATCACGCCCTGC 2340

Q E W **V** S F I V F D P F V E L F I T P C 780

ATCGTGGTCAACACGCTGTTCATGGCGCTCGACCACCACGACATGAACCCGGACATGGAG 2400

I V V N T L F M A L D H H D M N P D M E 800

CGGGCGCTCAAGAGCGGTAACTACTTCTTCACGGCGACGTTCGCGATCGAAGCGACGATG 2460

R A L K S G N Y F F T A T F A I E A T M 820

AAGCTGATCGCGATGAGCCCCAAGTGGTACTTCCAGGAAGGTTGGAACATTTTCGATTTC 2520

K L I A M S P K W Y F Q E G W N I F D F 840

ATCATCGTGGCCCTTTCGCTGCTCGAGCTCGGTCT**G**GAGGGCGTTCAGGGATTGTCAGTA 2580

-----------------------------------**R**------------------------

-----------------------------------**A**------------------------

I I V A L S L L E L G L E G V Q G L S V 860

TTACGTTCATTCCGTTTGCTTCGAGTGTTCAAGCTAGCAAAGTCGTGGCCAACGCTGAAC 2640

L R S F R L L R V F K L A K S W P T L N 880

TTACTCATTTCCATCATGGGCCGAACG**A**TGGG**C**GCGTTAGGTAATCTGACGTTTGTGCTC 2700

---------------------------**-**----**M**---------------------------

---------------------------**-**----**A**---------------------------

L L I S I M G R T **M** G A L G N L T F V L 900

TGCATTATCATCTTCATCTTTGCCGTGATGGGGATGCAGCTGTTCGGCAAGAACTACA**TC** 2760

C I I I F I F A V M G M Q L F G K N Y **I** 920

GACAACGTGGACCGCTTCCCGGACAAGGACCTGCCACGGTGGAACTTCACCGACTTCATG 2820

D N V D R F P D K D L P R W N F T D F M 940

CACTCATTCATGATCGTGTTCCGGGTGCTGTGCGGCGAGTGGATCGAATCCATGTGGGAC 2880

H S F M I V F R V L C G E W I E S M W D 960

TGCATGCTGGTGGGCGACGTGTCCTGCATTCCGTTCTTCTTGGCCACCGTAGTGATAGGA 2940

C M L V G D V S C I P F F L A T V V I G 980

AATTT**A**GTCGTCCTTAACCTTTTCTTAGCCTTGCTTTTGTCCAACTTTGGTTCCTCGAGT 3000

-----**W**------------------------------------------------------

-----**T**------------------------------------------------------

N **L** V V L N L F L A L L L S N F G S S S 1000

- L/**F** - - - - - - - - - - - - - - - - - - **HR/HRX(4)**

- **F** - - - - - - - - - - - - - - - - - -

TTGTCGGCGCCCACAGCCGACAACGAAACGAACAAGATCGCCGAGGCGTTCAAC**T**GGATA 3060

L S A P T A D N E T N K I A E A F N **W** I 1020

TCGCGCTTCTCCAACTGGATCAAGGCGAACATCGCGGCCGCGCTCAAGTTCGTGAAAAAC 3120

S R F S N W I K A N I A A A L K F V K N 1040

AAGTTAACAAGCCAGATTGCGTCCGTGCAGCCCGCAGGCAAAGGGGTATGTCCATGTATA 3180

K L T S Q I A S V Q P A G K G V C P C I 1060

TCTGCAGAGCATGGTGAAAATGAGC**T**GGAATTAACTCCAGATGACATCCTGGCCGACGGG 3240

S A E H G E N E **L** E L T P D D I L A D G 1080

CTGCTGAAAAAGGGCGTCAAGGAGCACAACCAGCTGGAGGTGGCGATCGGCGACGGGATG 3300

L L K K G V K E H N Q L E V A I G D G M 1100

GAGTTTACGATACACGGCGACCTCAAGAACAAGGGCAAGAAGAACAAGCAGCTGATGAAC 3360

E F T I H G D L K N K G K K N K Q L M N 1120

AATTCCAAGGTGATAGGCAATTCTATTAGTAATCATCAAGATAATAAGTTGGAGCACGAA 3420

N S K V I G N S I S N H Q D N K L E H E 1140

CTGAATCATAGGGGCATGTCCTTA**C**AGGACGATGATACTGCCAGTATAAAGTCCTATGGC 3480

L N H R G M S L **Q** D D D T A S I K S Y G 1160

AGTCACAAGAATCGCCCCTTCAAGGACGAAAGCCACAAGGGCAGTGCCGAAACGCTGGAG 3540

S H K N R P F K D E S H K G S A E T L E 1180

GGCGAAGAAAAGCGCGACGCCAGCA**A**GGAGGACCTAGGAATTGACGAAGAACTCGACGAC 3600

G E E K R D A S **K** E D L G I D E E L D D 1200

GAGTGCGAGGGTGAGGAGGGTCCTCTGGACGGGGAAATGATCATCCACGCGGAAGAGGAC 3660

E C E G E E G P L D G E M I I H A E E D 1220

GAGGTGATCGAGGACGCGCCGGCCGACTGCTTCCCGGACAACTGCTACAAGCGGTTCCCG 3720

E V I E D A P A D C F P D N C Y K R F P 1240

GC**A**CTGGCCGGCGA**C**GACGACGCGCC**G**TTCTGGCAGGGCTGGGGCAACCTGCGGCTCAAG 3780

--**R**-----------**Y**-----------**R**--------------------------------- **HR/HRX(5,6,7)**

--**G**-----------**T**-----------**A**---------------------------------

A L A G D D D A P F W Q G W G N L R L K 1260

ACGTTCCAGCTGATCGAGAACAAGTACTTCGAGACGGCCGTCATCACGATGATCCTGCTG 3840

T F Q L I E N K Y F E T A V I T M I L L 1280

AGTAGTTTGGCCCTGGCCCTCGAGGATGTGCACCTGCCGCACCGACCAATCTTGCAGGAC 3900

S S L A L A L E D V H L P H R P I L Q D 1300

GTCCTGTACTACATGGACAGGATATTCACGGTGATCTTTTTTTTAGAGATGTTGATCAAG 3960

V L Y Y M D R I F T V I F F L E M L I K 1320

TGGTTGGCGCTCGGCTTCCGGGTGTACTTTACG**A**ACGCCTGGTGCTGGCTCGATTTCATC 4020

W L A L G F R V Y F T **N** A W C W L D F I 1340

ATTGTGATGGTGTCCTTAATCAACTTCGTGGCTTCACTCTGTGGAGCGGGTGGTATTCAA 4080

I V M V S L I N F V A S L C G A G G I Q 1360

GCATTCAAAACTATGCGAACTCTTAGGGCACTGCGTCCGCTACGTGCCATGTCCCGTATG 4140

A F K T M R T L R A L R P L R A M S R M 1380

CAGGGTATGAGGGTTGTCGTCAATGCATTGGTACAGGCTATACCGTCCATCTTCAACGTG 4200

Q G M R V V V N A L V Q A I P S I F N V 1400

TTATTGGTGTGTTTGATCTTTTGGTTGATTTTCGCCAT**G**ATGGGCGTCCAGCTGTTTGCC 4260

L L V C L I F W L I F A **M** M G V Q L F A 1420

GGAAAGTACTTCAAGTGCGTCGACACGAACAAG**A**CGACACTGTCGCACGAGATCATCCCG 4320

G K Y F K C V D T N K **T** T L S H E I I P 1440

GACGTGAACGCGTGCATCGCGGAGAACTACACCTGGGAGAACTCCCCGATGAACTTTGAC 4380

D V N A C I A E N Y T W E N S P M N F D 1460

CACGTGGGGAAGGCCTACCTGTGTTTGTTCCAGGTGGCCACGTTCA**G**GGGATGGATCCAG 4440

H V G K A Y L C L F Q V A T F **R** G W I Q 1480

ATCATGAACGACGCGATCGACTCGCGGGACATCGGAAAGCAGCCCATCCGCGAAACCAAC 4500

I M N D A I D S R D I G K Q P I R E T N 1500

ATCTACATGTACTTGTACTTTGTGTTCTTCATCATCTTCGGATCGTTCTTCACGCTGAAC 4560

I Y M Y L Y F V F F I I F G S F F T L N 1520

CTCTTCATCGGTGTCATTATTGACAACTTTAACGAACAGAAGAAGA**A**GGCTGGGGGATCG 4620

L F I G V I I D N F N E Q K K **K** A G G S 1540

CTCGAGATGTTTATGACGGAGGACCAAAAAAAGTACTACAACGCAATGAAG**G**AGATGGGC 4680

L E M F M T E D Q K K Y Y N A M K **E** M G 1560

TCGAAGAAGCCACTGAAGGCCATTCCGCGGCCCAAG**T**GGCGACCACAAGCAATAGTGTTC 4740

------------------------------------**Y**-----------------------

------------------------------------**C**-----------------------

S K K P L K A I P R P K **W** R P Q A I V F 1580

- - - - - - - - - - - - **R/W** - - - - - - -

- - - - - - - - - - - - **R** - - - - - - - **HRX(8)**

GAAATCTGCACAAACAAAAAGTTCGACATGATCATCATGTTGTTCAT**G**GGCTTCAACATG 4800

E I C T N K K F D M I I M L F **M** G F N M 1600

TTGACGATGACGCTGGATCACTACAAGCAGACGGAAACGTTCAGCGCGGTGCTGGACTAC 4860

L T M T L D H Y K Q T E T F S A V L D Y 1620

CTGAACATGATCTTCATCTGTATCTTCAGTAGCGAGTGTCTGATGAAGATCTTCGCGCTG 4920

L N M I F I C I F S S E C L M K I F A L 1640

CGCTACCACTACTTTATCGAACCGTGGAACCTGTTCGATTTCGTCGTCGTCATCCTGTCC 4980

R Y H Y F I E P W N L F D F V V V I L S 1660

ATTTTGGGCCTGGTGCTGAGCGACCTGATCGAAAAGTACTTCGTCTCGCCGACGCTGCTC 5040

I L G L V L S D L I E K Y F V S P T L L 1680

CGTGTGGTGCGCGTGGCCAAGGTCGGTCGGGTGCTGCGTCTCGTCAAGGGCGCCAAGGGC 5100

R V V R V A K V G R V L R L V K G A K G 1700

ATCCGGACGTTGCTGTTTGCGCTGGCCATGTCGCTGCCGGCGCTGTTCAACATCTGTCTG 5160

I R T L L F A L A M S L P A L F N I C L 1720

CTGCTGTTCCTGGTGATGTTCATCTTCGCCATCTTCGG**A**ATGTCGTTCTTCATGCACGTG 5220

--------------------------------------**G**--------------------- **HR/HRX(9)**

L L F L V M F I F A I F G M S F F M H V 1740

AAGGACAAGAGCGGGCTGGACGACGTGTACAACTTCAAGACGTTCGGCCAGAGCATGATC 5280

K D K S G L D D V Y N F K T F G Q S M I 1760

CTGCTGTTTCAGATGTCAACGTCTGCGGGGTGGGACGGTGTGCTGGATGGTATCATCAAC 5340

L L F Q M S T S A G W D G V L D G I I N 1780

GAGGAGGACTGCCTGCCGCCGGAT**G**ACGACAAGGGTTACCC**C**GGGAACTGCGGGTCGGCG 5400

E E D C L P P D **D** D K G Y P G N C G S A 1800

ACGATCGGCATCACGTACCTGCTGGCATATCTGGTCATCAGTTTCCTGATCGTTATCAAC 5460

T I G I T Y L L A Y L V I S F L I V I N 1820

ATGTACATCGCTGTCATTCTCGAGAATTACTCGCAGGCCACGGAGGACGTGCAGGAGGGT 5520

M Y I A V I L E N Y S Q A T E D V Q E G 1840

CTGA**C**GGACGACGACTACGACATGTACTACGAGATCTGGCAGCAGTTCGATCCGGACGGT 5580

L **T** D D D Y D M Y Y E I W Q Q F D P D G 1860

ACGCAGTACATCCGGTACGACCAGCTGTCGGACTTTTTGGACGTGCTGGAACCGCCGCTG 5640

T Q Y I R Y D Q L S D F L D V L E P P L 1880

CAGATTCACAAACCGAACAAGTACAAGATCATCTCGATGGACATTCCGATCTGTCGCGGC 5700

Q I H K P N K Y K I I S M D I P I C R G 1900

GACATGATGTTCTGCGTGGACATTCTGGACGCGCTGACGAAGGACTTCTTCGCGCGGAAG 5760

D M M F C V D I L D A L T K D F F A R K 1920

GGCAACCCGATCGAGGACAGTGCCGAGATGGGTG**A**GGTCCAGCAGCGGCCGGACGAGGTC 5820

G N P I E D S A E M G **E** V Q Q R P D E V 1940

GGTTACGAGCCGGTTTCGTCGACGTTGTGGCGCCAACGGGAGGAGTACTGCGCGCGGTTG 5880

G Y E P V S S T L W R Q R E E Y C A R L 1960

ATACAGCACGCGTACCGGAACTTTAAGGAACGAGGCGGTGTTGGTGGCGGCGGCGGCGGT 5940

I Q H A Y R N F K E R G G V G G G G G G 1980

GGAGGTGGTGGAGGAGGAGGTGGTGGCGAAGGTGCCGGAGATGACACCGACGCCGATGCC 6000

G G G G G G G G G E G A G D D T D A D A 2000

TGTGATAACGAGCCCGGGATCGGGAGTCCCGGCGCGGTCAGCGGCGGTGGCGGCAGCATC 6060

C D N E P G I G S P G A V S G G G G S I 2020

GCCGGCGGAGGCTCCCAGGCTAACCTAGGGCCGCCGTCACCCAAAGAATCGCCCGATGGC 6120

A G G G S Q A N L G P P S P K E S P D G 2040

AATAATGATCCTCAAGGTCGTCAAACGGCCGTCCTAGTAGAAAGTGATGGATTTGTAACT 6180

N N D P Q G R Q T A V L V E S D G F V T 2060

AAAAACGGTCACCGTGTCGTGATACACTCACGATCGCCAAGTATAACTTCACGATCGGCG 6240

K N G H R V V I H S R S P S I T S R S A 2080

GATGTCTGAGCCAGGCCTCGCCCCCCTCCCTCTGATTCAGATTCAGAAGCACGACAGAAA 6300

D V * 2100

TAATATTTTAAAGATTAAGAAAAATACTTAAAAAACAAAACCG***AAAAAAAAAAAAAAAAA*** 6300

***AAAAAAAAAAA***

**Figure S1.** Alignment of the cDNA/deduced amino acid sequences in mosquitoes. The cDNA/deduced amino acid sequences of the full length *Cx. quinquefasciatus* sodium channel cDNA were compared among S-Lab, HAmCq^G0^, and HAmCq^G8^ (Accession numbers: JN695777, JN695778, JN695779). The nucleotides/deduced amino acids are numbered from transcription start point (*tsp*)/translation start codon (*tsc*) as +1. The *tsp*/*tsc* and a polyadenylation signal are in bold and italics. The nonsynonymous codons and corresponding amino acid substitutions are highlighted and polymorphism codes are underlined. The synonymous codons are highlighted and polymorphism codes are doubly underlined. In this pilot study, the sequence was generated from a pool of mosquito cDNAs for each of three mosquito strains, with a total of 9 complete sodium channel cDNA sequences being analyzed for each. Unlike the other nonsynonymous and synonymous mutations, SNPs of which were only presented in the resistant HAmCq^G0^ and HAmCq^G8^ strains, both homozygous susceptible alleles and heterozygous alleles at the codons of the nonsynonymous A^109^S and synonymous G^1752^G were presented in the susceptible S-lab mosquitoes, only the homozygous polymorphic alleles was observed in the highly resistant HAmCq^G8^ strain.
