# Supplementary material for: Evolutionary Adaptation of the Amino Acid and Codon Usage of the Mosquito Sodium Channel following Insecticide Selection in the Field Mosquitoes
Source: PLoS One. 2012 Oct 17;7(10):e47609. doi: 10.1371/journal.pone.0047609 (PMC3474719; doi:10.1371/journal.pone.0047609)
Supplement: Table S1 — Toxicity of permethrin to the S-Lab strain before and after permethrin selection. aNumber of selected fourth instar larvae bLC50 values in ppm c95% confidence interval, toxicity of permethrin is considered significantly different when the 95% CI fail to overlap dRR: LC50 of selected generations/LC50 of the S-Lab strain eParental strain – i.e., S-Lab (DOC) [file pone.0047609.s002.doc]

**Table S1.** Toxicity of permethrin to the S-Lab strain before and after permethrin selection.

| Strains | Selected generation | Selecting concentration (ppm) | n^a^ | Survival (%) | LC_50_^b^ (CI)^c^  (ppm) | RR^d^ | Slope (SE) |
| --- | --- | --- | --- | --- | --- | --- | --- |
| S-Lab | G0^e^ |  |  |  | 0.0080 (0.006-0.0 | 1 | 4.0 (0.9) |
|  | G1 | 0.005 | ~6000 | ~30% | 0.0076 (0.005-0.01) | 0.95 | 3.9 (1.0) |
|  | G2 | 0.006 | ~5000 | ~35% | 0.0080 (0.006-0.01) | 1 | 4.0 (0.9 |
|  | G3 | 0.005 | ~5000 | ~30% | 0.0082 (0.005-0.01) | 1 | 4.0 (0.9 |
|  | G4 | 0.005 | ~6000 | ~30% | 0.0080 (0.006-0.01) | 1 | 3.8 (0.7) |
|  | G5 | 0.005 | ~6500 | ~35% | 0.0075 (0.006-0.01) | 0.94 | 3.8 (0.8) |

^a^ Number of selected fourth instar larvae

^b^ LC_50_ values in ppm

^c^ 95% confidence interval, toxicity of permethrin is considered significantly different when the 95% CI fail to overlap

^d^ RR: LC_50_ of selected generations /LC_50_ of the S-Lab strain

^e^Parental strain – i.e., S-Lab
