# Supplementary material for: Evolutionary Adaptation of the Amino Acid and Codon Usage of the Mosquito Sodium Channel following Insecticide Selection in the Field Mosquitoes
Source: PLoS One. 2012 Oct 17;7(10):e47609. doi: 10.1371/journal.pone.0047609 (PMC3474719; doi:10.1371/journal.pone.0047609)
Supplement: Table S2 — Oligonucleotide primers* used for amplifying the sodium channel cDNA, qRT-PCR reactions and SNP (single nucleotide polymorphism) determination. *Designation of oligonucleotide mixtures: R = A+G; Y = C+T; K = G+T. (DOC) [file pone.0047609.s003.doc]

Table S2. Oligonucleotide primers^*^ used for amplifying the sodium channel cDNA, qRT-PCR reactions and SNP (single nucleotide polymorphism) determination.

| Primer name | Function | Primer sequence (5’ to 3’) | Primer Location (nt) |
| --- | --- | --- | --- |
| KDR S16 | cDNA fragment 1 and full length amplification | TGTTGGCCATATAGACAATGACCGA | -17 to 8 |
| KDR AS34 | cDNA fragment 1 amplification and 5’ RACE | GTAATACTGACAATCCCTGAACGC | 2584 to 2561 |
| PG_KDR S4 | cDNA fragment 2 amplification | GCGGTAACTACTTCTTCACGGC | 2414 to 2435 |
| KDR AS02 | cDNA fragment 2 amplification | CCAKCCYCTRAAKGTGGCYACTTG | 4411 to 4434 |
| KDR S03 | cDNA fragment 3 amplification and 3’RACE | TGAACTTYGACCACGTGGGG | 4370 to 4389 |
| KDR AS09 | cDNA fragment 3 and full length amplification | GCTTCTGAATCTGAATCAGAGGGAG | 6290 to 6266 |
| AP1 | 5’ and 3’ RACE | CCATCCTAATACGACTCACTATAGGGC | Adapter |
| Oligo(dT) | 1^st^ strained cDNA | TAATACGACTCACTATAGGGAGATTTTTTTTTTTTTTTT | Tomita and Scott, 1995 |
| Cx_SNP 2 | SNaP determination | GCCACCGTAGTGATAGGAAATTT | 2923 to 2945 |
| Cx_SNP 4 | SNaP determination | CTCGAGGATATTGACGCTTTTTAC | 301 to 324 |
| Cx_SNP 6 | SNaP determination | TGAAGGCCATTCCGCGGCCCAAG | 4694 to 4716 |
| Cx_SNP 12 | SNaP determination | CTTTCGCTGCTCGAGCTCGGTCT | 2533 to 2555 |
| Cx_SNP 13 | SNaP determination | TCCATCATGGGCCGAACGATGGG | 2650 to 2672 |
| Cx_SNP 14 | SNaP determination | AACTGCTACAAGCGGTTCCCGGC | 3700 to 3722 |
| Cx_SNP 15 | SNaP determination | GGTTCCCGGCRCTGGCCGGCGA | 3713 to 3734 |
| Cx_SNP 16 | SNaP determination | TGGCCGGCGAYGACGACGCGCC | 3725 to 3746 |
| Cx_SNP 18 | SNaP determination | ATGTTCATCTTCGCCATCTTCGG | 5176 to 5198 |

^*^Designation of oligonucleotide mixtures: R = A+G; Y = C+T; K = G + T.
